# Reciprocal Regulation of Metabolic Reprogramming and Epigenetic Modifications in Cancer

**DOI:** 10.3389/fgene.2018.00394

**Published:** 2018-09-19

**Authors:** Xilan Yu, Rui Ma, Yinsheng Wu, Yansheng Zhai, Shanshan Li

**Affiliations:** State Key Laboratory of Biocatalysis and Enzyme Engineering, Hubei Key Laboratory of Industrial Biotechnology, College of Life Sciences, Hubei University, Wuhan, China

**Keywords:** metabolism, epigenetic modifications, histone acetylation, histone methylation, DNA methylation, transcription, tumorigenesis

## Abstract

Cancer cells reprogram their metabolism to meet their demands for survival and proliferation. The metabolic plasticity of tumor cells help them adjust to changes in the availability and utilization of nutrients in the microenvironment. Recent studies revealed that many metabolites and metabolic enzymes have non-metabolic functions contributing to tumorigenesis. One major function is regulating epigenetic modifications to facilitate appropriate responses to environmental cues. Accumulating evidence showed that epigenetic modifications could in turn alter metabolism in tumors. Although a comprehensive understanding of the reciprocal connection between metabolic and epigenetic rewiring in cancer is lacking, some conceptual advances have been made. Understanding the link between metabolism and epigenetic modifications in cancer cells will shed lights on the development of more effective cancer therapies.

## Introduction

One hallmark of tumor cells is their rewired metabolism to meet the requirement for macromolecular biosynthesis, survival, and proliferation (Yu and Li, 2017). For example, cancer cells prefer aerobic glycolysis rather than oxidative phosphorylation to generate energy, a phenomenon called “Warburg effect” (Yu and Li, 2017). However, as aerobic glycolysis is a less efficient way to provide energy, it remains unclear why tumor cells prefer aerobic glycolysis. It has been proposed that the accelerated aerobic glycolysis in cancer cells could facilitate the accumulation of precursors for macromolecules, i.e., nucleic acids, proteins, lipids to fulfill their high anabolic demands (Vander Heiden et al., 2009). Recent studies revealed that many metabolic enzymes can translocate into the nucleus and have non-metabolic functions in tumorigenesis (Yu and Li, 2017). One important non-metabolic function is regulating epigenetic modifications and gene expression. The reprogrammed metabolism in cancer cells may lead to different epigenetic landscapes that contribute to malignant tumor growth.

Epigenetics is defined as heritable changes in gene expression independent of mutations in genomic DNA. It originally includes histone post-translational modifications such as acetylation, methylation, ubiquitination, phosphorylation, SUMOylation and DNA modifications. With the development of proteomics and mass spectrometry technology, the repertoire of chromatin modifications is expanding with more epigenetic modifications identified such as acylation (crotonylation, succinylation, propionylation, β-hydroxybutyrylation), *O*-linked *N*-acetylglucosamine (*O*-GlcNAcylation) and RNA modifications (Huang et al., 2014). These modifications play important roles in dictating chromatin structure, regulating gene transcription and contributing to tumorigenesis. Cancer cells have distinct epigenetic modification patterns with their normal counterparts and some epigenetic modification alterations frequently occur at oncogenes and/or tumor-suppressor genes to facilitate malignant transformation and tumorigenesis (Feinberg and Vogelstein, 1983; Esteller, 2008). For example, DNA hypomethylation was found in genes of human cancers compared with their normal tissue counterparts (Feinberg and Vogelstein, 1983). Unlike genetic mutations, epigenetic modifications are always reversible, enabling the development of chemical interventions for cancer therapy. In fact, several drugs targeting epigenetic modifications have been approved by the Food and Drug Administration (FDA) and a large number of compounds are under preclinical or clinical trials (Gao et al., 2017).

Cellular metabolism and epigenetic modifications interact with one another and are regulated in a reciprocal manner. Most chromatin post-translational modifications, such as phosphorylation, acetylation, methylation, acylation, and O-linked *N*-acetylglucosamine modification (O-GlcNAcylation), require metabolites as substrates or cofactors, such as acetyl-CoA for histone acetylation and S-adenosylmethionine (SAM) for histone and DNA methylation (Kinnaird et al., 2016). Altered epigenetic landscape also changes the expression of metabolic genes and/or the activity of metabolic enzymes, which in turn rewires the cellular metabolism in tumors. The intimate connection between metabolism and epigenetic modifications contributes to tumor initiation and progression.

## Metabolic Regulation of Epigenetic Modifications

### Modulation of Epigenetic Modifications by Metabolites

Most chromatin modifying enzymes use intermediary metabolites as cofactors or substrates and thus their activity is regulated by the availability of these metabolites. In the following sections, we will first describe the metabolism of four common intermediates (acetyl-CoA, SAM, α-ketoglutarate, NAD^+^). Then we will discuss the effect of metabolites from glycolysis, tricarboxylic acid (TCA) cycle, and fatty acids metabolism on chromatin modifications.

#### Acetyl-CoA Metabolism and Histone Acetylation

**FIGURE 1:**
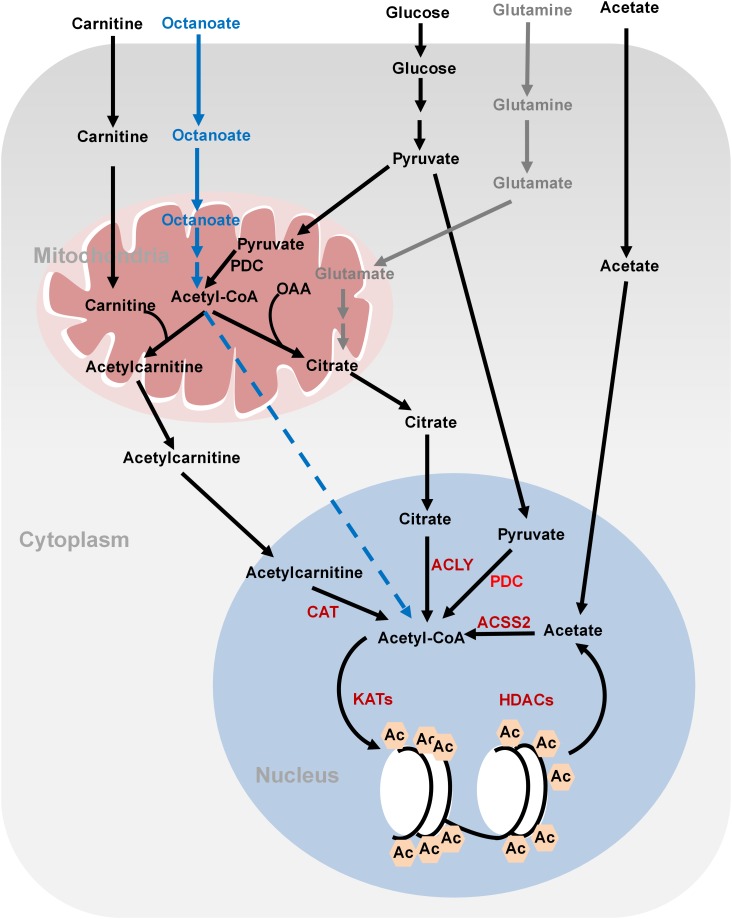
Metabolism of acetyl-CoA and histone acetylation. Glucose-derived pyruvate is metabolized to acetyl-CoA by PDC in the mitochondria. Mitochondrial acetyl-CoA needs to be converted to citrate or acetylcarnitine in order to be exported into cytoplasm and nucleus. In the nucleus, acetyl-CoA is regenerated from citrate and acetylcarnitine by ATP-citrate lyase (ACLY) and carnitine acetyltransferase (CAT), respectively, for histone acetylation. Nucleus acetyl-CoA can also be produced from glucose-derived pyruvate by nucleus PDC. Fatty acids (octanoate) can be oxidized to produce acetyl-CoA in the mitochondria but it is unknown how it is transported into the nucleus. ACSS2 synthesizes acetyl-CoA from acetate, which is derived from the media or deacetylation reactions. Glutamine can be used to synthesize citrate through reductive carboxylation in the mitochondria. Citrate can then be translocated into the nucleus to generate nucleus acetyl-CoA. OAA, oxaloacetate; ACLY, ATP-citrate lyase; PDC, pyruvate dehydrogenase complex; ACSS2, acetyl-CoA synthetase short chain family member 2; CAT, carnitine acetyltransferase; Ac, acetylation.

Histone acetylation is performed by lysine acetyltransferases (KATs) that transfer the acetyl group from acetyl-CoA to histones. Acetylation neutralizes the positive charges of lysine residues on histones, which eliminates the electrostatic interaction between histones and DNA, leading to a less compact chromatin structure permissive for gene transcription. Histone acetylation is sensitive to changes of global acetyl-CoA levels. As the acetyl group donor, acetyl-CoA is generated from three major sources: glucose, fatty acids, and acetate (Sebastian and Mostoslavsky, 2017; **Figure 1**). Glucose is an important source for acetyl-CoA and declines in glycolytic flux significantly reduce the intracellular levels of acetyl-CoA. As a result, the levels of about 40% of the identified histone acetylation sites are reduced, including H3K9ac, H3K14ac, H3K18ac, H4K8ac, H4K12ac, and H4K16ac (Cluntun et al., 2015). The glycolysis product, pyruvate is fueled to acetyl-CoA synthesis by pyruvate dehydrogenase complex (PDC) in the mitochondria. However, as acetyl-CoA cannot cross the organelle membrane, acetyl-CoA is translocated out of mitochondria in the form of citrate, which then diffuses to the nucleus where ATP-citrate lyase (ACLY) cleaves it to produce nuclear acetyl-CoA for histone acetylation. In addition to citrate-derived acetyl-CoA by nuclear ACLY, acetyl-CoA can be translocated into the nucleus for histone acetylation by carnitine/acetylcarnitine transport system in cancer cells (Madiraju et al., 2009). The excessive mitochondrial acetyl-CoA, produced from pyruvate by PDC, is converted to acetylcarnitine by carnitine acetyltransferase (CAT; Madiraju et al., 2009). Acetylcarnitine is transported into cytoplasm by carnitine/acylcarnitine translocase and then to nuclear matrix, where a nuclear CAT converts acetylcarnitine to acetyl-CoA, which is used for histone acetylation (**Figure 1**; Madiraju et al., 2009). Nuclear acetyl-CoA can also be produced from glucose-derived pyruvate by translocation of metabolic enzymes to the nucleus. For example, PDC, originally found in the mitochondrial matrix, can translocate from the mitochondria into the nucleus where it generates high concentrations of nuclear acetyl-CoA from pyruvate and increases histone acetylation to activate the expression of S phase regulators (**Figure 1**; Sutendra et al., 2014).

Fatty acids are also a bona fide source of carbon for histone acetylation, contributing up to 90% of certain histone acetylation markers, i.e., H3K9ac, in immortalized hepatocytes (McDonnell et al., 2016). Feeding cells with medium-chain fatty acids such as octanoate, hexanoate, decanoate, and dodecanoate increases acetyl-CoA concentrations in the mitochondria via fatty acid oxidation, which then elevates the global levels of histone acetylation and activates the transcription of genes involved in fatty acid metabolism (McDonnell et al., 2016). Fatty acids are the predominant contributor to global acetyl-CoA and histone acetylation even in the presence of glucose (McDonnell et al., 2016). Interestingly, ACLY is not required for lipid-derived acetyl-CoA production and global histone acetylation (**Figure 1**). It remains unclear how mitochondrial lipid-derived acetyl-CoA is translocated into the nucleus.

Cancer cells also synthesize acetyl-CoA from acetate by acetyl-CoA synthetase short chain family member 2 (ACSS2) (**Figure 1**; Zhao et al., 2016). Acetate could not only come from medium but also from the product of histone deacetylation. Under hypoxia conditions, ACSS2 translocates into the nucleus where it synthesizes nuclear acetyl-CoA from acetate for histone acetylation (Schug et al., 2015).

For cancer cells that cannot undergo normal oxidative phosphorylation in the mitochondria, they use glutamine-dependent reductive carboxylation as the major pathway to generate citrate and acetyl-CoA (**Figure 1**; Mullen et al., 2011). Glutamine is an important respiratory substrate in cancer cells, providing energy and carbon source for cancer growth. Inhibition of glutamine catabolism has been shown to become a promising cancer therapy strategy (Momcilovic et al., 2018). Glutamine-derived α-ketoglutarate (α-KG) is converted to isocitrate by isocitrate dehydrogenase (IDH), which is then converted to citrate and concomitant acetyl-CoA. This glutamine-dependent pathway is the dominant metabolism for acetyl-CoA production in rapidly growing malignant cells containing mutations in electron transport chain (ETC) or in cells subject to acute pharmacological ETC inhibition (Mullen et al., 2011). The multiple ways for cancer cells to synthesize acetyl-CoA reflect their metabolism plasticity in response to nutrient changes in the microenvironment.

#### SAM Metabolism and DNA and Histone Methylation

**FIGURE 2:**
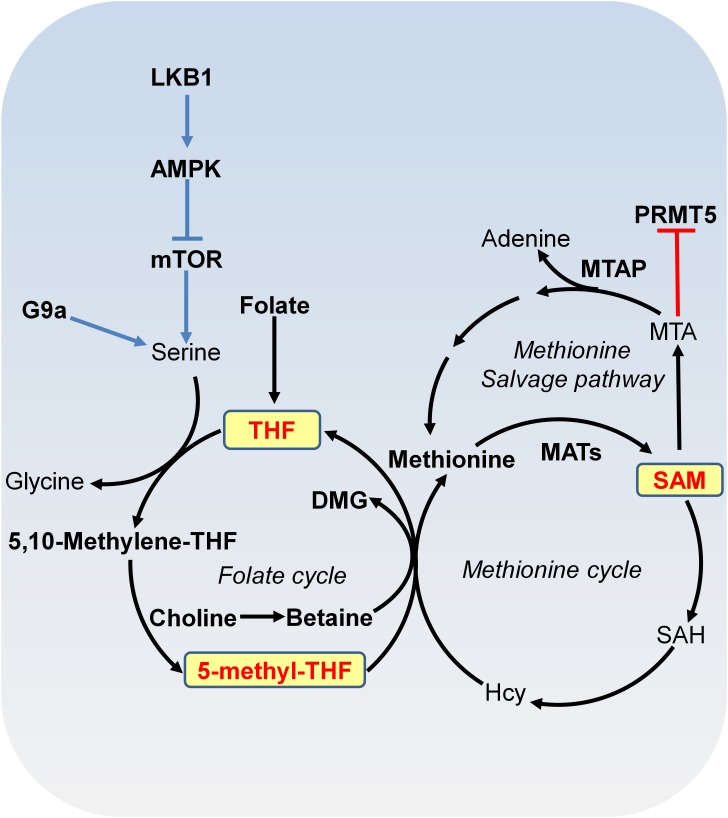
Metabolism of SAM. SAM is synthesized from methionine and ATP by methionine adenosyltransferase (MAT). In methylation reactions, SAM is sequentially converted to S-adenosylhomocysteine (SAH), homocysteine (Hcy) and methionine with 5-methyl-tetrahydrofolate (THF) or with betaine as the methyl donor. Serine-glycine metabolism provides one-carbon unit to the folate cycle. Serine biosynthesis is controlled by LKB-AMPK-mTOR pathway. In the methionine salvage pathway, SAM is converted to 5′-methylthioadenosine (MTA), which is salvaged back for SAM generation. MTA inhibits the activity of PRMT5. MAT, methionine adenosyltransferase; MTA, 5′-methylthioadenosine; THF, tetrafolate; LKB1, liver kinase B1; mTOR, mammalian target of rapamycin complex; SAH, S-adenosylhomocysteine; Hcy, homocysteine; MTAP, MTA phosphorylase; DMG, dimethylglycine; PRMT5, arginine methyltransferase 5.

DNA methylation and histone methylation are catalyzed by methyltransferases with SAM as the methyl donor. SAM is derived from combined activities of one-carbon metabolism and methionine metabolism through a vitamin-dependent metabolic cycle (**Figure 2**; Schvartzman et al., 2018). SAM is synthesized by methionine adenosyltransferase (MAT) from methionine and ATP. Knockout of *MAT1A* in mice has been shown to reduce hepatic SAM levels and lead to misregulation of genes involved in the metabolism of lipids and carbohydrates and spontaneous development of hepatocellular carcinoma (HCC; Martinez-Chantar et al., 2002). In histone and DNA methylation reactions, SAM is converted to S-adenosylhomocysteine (SAH), which can then be hydrolyzed to homocysteine (Hcy). Hcy can be recycled to methionine by methionine synthase (MS) with 5-methyl-tetrahydrofolate (5-methyl-THF) as the methyl donor or by betaine-homocysteine methyltransferase (BHMT) with betaine as the methyl donor. 5-methyl-THF is derived from THF by serine, glycine and one-carbon metabolism or folate cycle (**Figure 2**). Enzymes that catalyze serine, glycine and folate metabolism are differentially upregulated in a broad spectrum of tumors (Locasale et al., 2011; Possemato et al., 2011). For example, phosphoglycerate dehydrogenase (PHGDH), the first and rate-limiting enzyme in glucose-derived serine biosynthesis, is upregulated in breast cancer and melanoma due to amplification of its gene copy (Locasale et al., 2011). Further efforts are required to determine the impact of PHGDH amplification on DNA and histone methylation in cancer cells.

S-adenosylmethionine can also be regenerated by methionine salvage pathway, where SAM is decarboxylated to form 5′-methylthioadenosine (MTA), which is then salvaged back to methionine and SAM (**Figure 2**). MTA phosphorylase (MTAP) cleaves MTA to generate precursors for methionine salvage pathway. MTAP is ubiquitously expressed in normal tissues; however, because *MTAP* gene locates close to tumor suppressor gene *CDKN2A*, *MTAP* homologous deletion occurs frequently in cancers such as 40% in glioblastomas; 25% in melanomas, urothelial carcinomas and pancreatic adenocarcinomas; 15% in non-small cell lung carcinomas (Kryukov et al., 2016). MTA specifically inhibits the activity of arginine methyltransferase 5 (PRMT5) to catalyze symmetric dimethyl histone H4 arginine 3 (H4R3me2s) and loss of MTAP confers specific vulnerability to PRMT5 inhibition (Kryukov et al., 2016).

Due to the tight connection between SAM availability and DNA and histone methylation, factors that perturb SAM levels or SAM/SAH ratio could determine DNA and histone methylation status (Mentch et al., 2015). These factors include intermediary metabolites or cofactors involved in SAM metabolism (methionine, vitamins, particularly, folate, vitamins B6, and B12) and one-carbon metabolism (serine, glycine, and threonine). Modulation of methionine in diet leads to changes in H3K4me3, altered gene expression, and feedback control of one-carbon metabolism in the liver (Mentch et al., 2015; Dai et al., 2018). Reduced methionine uptake via knockdown of methionine transporter Lat1 impairs the activity of H3K27 methyltransferase EZH2 and inhibits tumor growth (Dann et al., 2015). Depletion of threonine or knockdown of threonine dehydrogenase decreases the ratio of SAM/SAH as well as the cellular levels of H3K4 di- and trimethylation (H3K4me2, H3K4me3), leading to reduced cell growth (Shyh-Chang et al., 2013). In addition, SAM availability regulates gene expression via DNA methylation. SAM treatment induces DNA hypermethylation at the promoter of vascular endothelial growth factor-C (VEGF-C) and subsequently reduces VEGF-C expression, which inhibits gastric cancer cell growth and tumorigenesis (Da et al., 2014).

#### α-Ketoglutarate (α-KG) Metabolism and DNA and Histone Demethylation

DNA and histone methylation can be actively removed by demethylases. There are two major classes of demethylases: flavin adenine dinucleotide (FAD)-dependent LSD demethylases and α-KG-dependent JmjC family demethylases. The LSD family of histone demethylases (LSD1 and LSD2) use oxygen to remove methyl groups from mono- or dimethylated histones in a FAD-dependent manner. JmjC demethylases use oxygen and α-KG as substrates, producing succinate and CO_2_. JmjC demethylases include a diverse family of enzymes responsible for histone demethylation, DNA 5-methyl-cytosine hydroxylation, RNA *N*^6^-methyladenosine (m^6^A) demethylation, etc.

**FIGURE 3:**
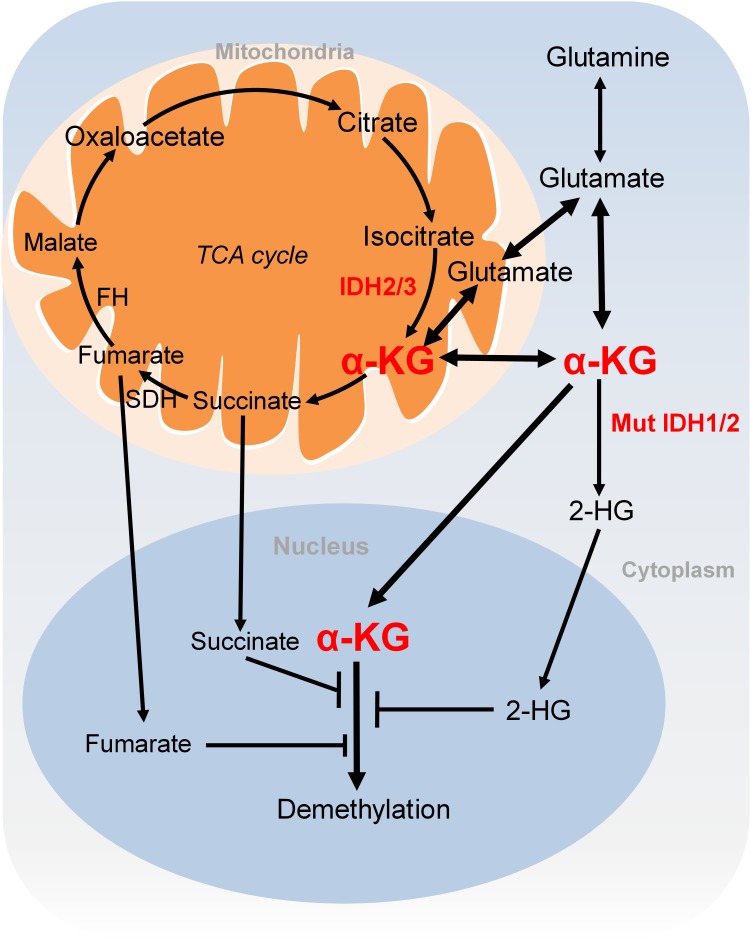
Metabolism of α-KG. α-KG is synthesized from the TCA cycle or transamination of glutamate derived from glutamine. α-KG can be metabolized to succinate and fumarate in the TCA cycle. Succinate and fumarate are competitive inhibitors of α-KG-dependent demethylases. α-KG can also be converted to 2-HG by mutated isocitrate dehydrogenase 1 and 2 (IDH1 and IDH2). 2-HG is a competitive inhibitor of α-KG-dependent demethylases. α-KG, α-ketoglutarate; 2-HG, 2-hydroxyglutarate; Mut IDH1/2, mutated isocitrate dehydrogenase 1 and 2.

α-KG is either generated as an intermediary metabolite of the TCA cycle or produced by transamination of glutamate derived from glutamine (**Figure 3**). Glutamine is a major source for α-KG production and glutamine deprivation triggers accumulation of H3K27me3 and H4K20me3 in cultured cells, consistent with impaired α-KG-dependent histone demethylation (Carey et al., 2015). In proliferative cells, glutamine uptake is increased to promote α-KG production and accumulation (Wise and Thompson, 2010). Glutamine metabolism is pretty high in cancer cells, which may lead to regional depletion of glutamine, i.e., core region of solid tumours displayed glutamine deficiency (Pan et al., 2016). Regional depletion of glutamine may cause cancer cell dedifferentiation and drug resistance in part by decreasing the intracellular α-KG and inhibiting KDM6B-mediated H3K27 demethylation (Pan et al., 2016). Interestingly, H3K4me3 level is not responsive to changes of intracellular α-KG concentrations (Hwang et al., 2016). It remains unclear about how the specificity of chromatin modifications is determined by α-KG availability.

#### NAD^+^ Metabolism and Chromatin Deacetylation

**FIGURE 4:**
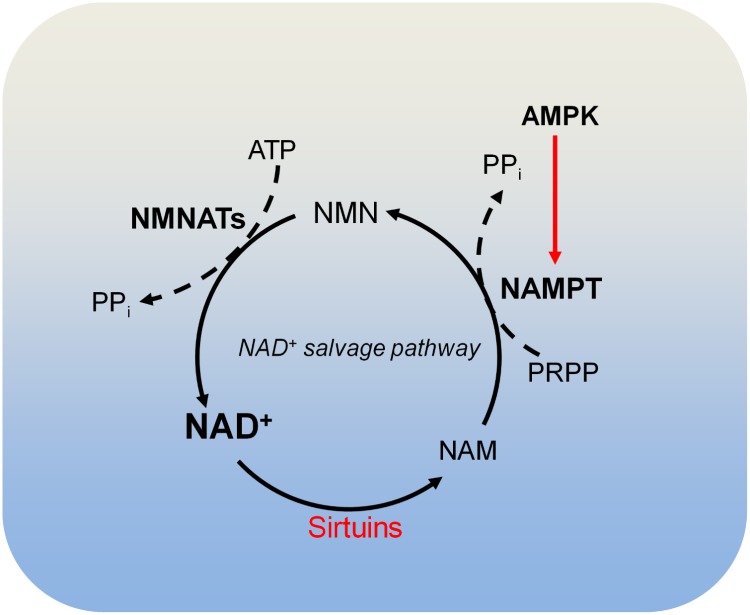
Metabolism of NAD^+^. Sirtuins consume NAD^+^ and produce nicotinamide (NAM). NAM is recycled to produce NAD^+^ by the NAD^+^ salvage pathway. In the NAD^+^ salvage pathway, NAM is converted to nicotinamide mononucleotide (NMN) by nicotinamide phosphoribosyltransferase (NAMPT), and NMN is then converted to NAD^+^ by nicotinamide mononucleotide adenylyltransferases (NMNATs). AMPK is required for NAMPT expression and thus controls intracellular NAD^+^. NAM, nicotinamide; NAMPT, nicotinamide phosphoribosyltransferase; NMNATs, nicotinamide mononucleotide adenylyltransferases; PRPP, phosphoribosyl pyrophosphate.

There are four classes of histone deacetylases (HDAC classes I, II, III, and IV) that remove acetyl moieties from histone lysine residues (Schvartzman et al., 2018). Class I, class II, and class IV HDACs are zinc-dependent enzymes. Class III HDACs, also known as sirtuins, deacetylate histone lysine residues using NAD^+^ as the co-substrate, which is cleaved to acyl-ADP-ribose and nicotinamide (NAM) (**Figure 4**; Schvartzman et al., 2018). NAM is recycled to produce NAD^+^ by NAD^+^ salvage pathway, which is known to be dominant for intracellular NAD^+^ biosynthesis in many cells and tissues (Khan et al., 2007; Chini et al., 2014). In the NAD^+^ salvage pathway, nicotinamide phosphoribosyltransferase (NAMPT) converts NAM to nicotinamide mononucleotide (NMN), which is then converted to NAD^+^ by nicotinamide mononucleotide adenylyltransferases (NMNAT) (**Figure 4**). Among these enzymes, NAMPT is the rate-limiting enzyme in NAD^+^ salvage pathway and is upregulated in tumors such as colorectal cancers (Van Beijnum et al., 2002). Due to its role in maintaining NAD^+^ levels in tumors, NAMPT has become an attractive target for cancer treatment and inhibition of NAMPT impairs pancreatic tumor growth (Chini et al., 2014). The NAMPT inhibitor, APO866 has been used in Phase II clinical trials against several human cancers (Khan et al., 2007).

During glycolysis, NAD^+^ is converted to NADH, leading to reduced NAD^+^/NADH ratio and downregulated activity of sirtuins. Therefore, it is possible that increased aerobic glycolysis in cancer may reduce the activity of sirtuins by decreasing NAD^+^/NADH ratio, leading to histone hyperacetylation, decondensed chromatin structure and dysregulation of gene expression (Yun et al., 2012). In addition, NAD^+^ metabolism is regulated by nutrients in diet. Calorie restriction potentially increases NAD^+^/NADH and enhances protein deacetylation, whereas high-fat diet decreases NAD^+^ levels and sirtuins’ activity.

There are three NAD^+^-producing NMNATs (NMNAT1, NMNAT2, and NMNAT3) with distinct subcellular localizations. NMNAT1 exists in the nucleus and is responsible for nuclear production of NAD^+^; NMNAT2 and NMNAT3 are localized in the Golgi complex and mitochondria, respectively (Berger et al., 2005). The differential localization of these NMNATs may be responsible for compartmentalized NAD^+^ inside cells (Berger et al., 2005).

#### Impact of Glycolytic Metabolites on Chromatin Modifications

Glycolysis provides several intermediary metabolites that regulate chromatin modifications. In addition to providing acetyl-CoA for histone acetylation, glycolysis enhances histone acetylation by inhibiting the reverse process. The product of glycolysis, pyruvate acts as an inhibitor for histone deacetylase 1 and 3 (HDAC1/3) and thus promotes histone acetylation (Thangaraju et al., 2006). Lactate, the end product of aerobic glycolysis has also been reported to inhibit the activity of HDACs (Latham et al., 2012).

Glycolysis is required for pyruvate kinase-mediated histone H3T11 phosphorylation and glycolytic metabolites fructose 1, 6-biphosphate (FBP) and phosphoenolpyruvate (PEP) function as the cofactor and substrate, respectively (Li et al., 2015; Yu et al., 2017). In addition, glycolysis is required for H4K8 lysine 2-hydroxyisobutyrylation (H4K8_hib_), which in turn regulates the transcription of genes involved in carbon transport and metabolism (Huang et al., 2017).

#### Impact of Tricarboxylic Acid (TCA) Cycle on Histone Demethylation

Another extensively studied metabolism that regulates chromatin modifications is the tricarboxylic acid (TCA) cycle. As described earlier, the TCA intermediary metabolite α-KG serves as the substrate for JmjC demethylases. Other TCA intermediary metabolites especially for those structurally related metabolites including succinate and fumarate are two competitive inhibitors of α-KG-dependent demethylases (**Figure 3**; Kinnaird et al., 2016). Two enzymes, fumarate hydratase (FH) and succinate dehydrogenase (SDH) mediate the metabolism of fumarate and succinate in the TCA cycle and are considered as tumor suppressors. Mutations of these two enzymes lead to accumulation of fumarate and succinate, resulting in enzymatic inhibition of multiple α-KG-dependent dioxygenases and subsequent genome-wide alterations of DNA and histone methylation, which eventually contribute to tumorigenesis (Xiao et al., 2012).

The oncometabolite D-2-hydroxyglutarate (D2-HG) is another competitive inhibitor of α-KG-dependent enzymes. Due to its structural similarity to α-KG, D2-HG outcompetes α-KG for binding to histone demethylases, i.e., JHDM. D2-HG is typically maintained at low levels in normal cells but is significantly elevated in tumor cells, i.e., glioma and melanoma (Dang et al., 2009; Gross et al., 2010). Excess accumulation of D2-HG can lead to an elevated risk of malignant brain tumors in patients (Schvartzman et al., 2018). D2-HG is derived from α-KG by mutated isocitrate dehydrogenase 1 and 2 (IDH1 and IDH2), while wild type IDHs catalyze the interconversion of isocitrate and α-KG (**Figure 3**; Dang et al., 2009). Mutations of IDH1 and IDH2 lead to accumulation of D2-HG, which increased the levels of histone methylation, i.e., H3K9me3 and H3K27me3 (Lu et al., 2012; Turcan et al., 2012). Moreover, D2-HG enhances gene silencing by inhibiting H3K36 histone demethylases (Janke et al., 2017). Mutations in IDH1 and IDH2 have been observed in tumors such as acute myelogenous leukemia (AML), grade II-III glioblastoma, chondrosarcoma and have been implied in promoting tumorigenesis by altering DNA and histone methylation status (Schvartzman et al., 2018).

#### Impact of Fatty Acid Metabolism on Histone Modifications

Fatty acid oxidation is an important source for acetyl-CoA and histone acetylation. As *de novo* fatty acid synthesis and histone acetylation use the same pool of acetyl-CoA, blocking fatty acid synthesis by reducing the expression of acetyl-CoA carboxylase (ACC1), which catalyzes the carboxylation of acetyl-CoA to malonyl-CoA during fatty acid biosynthesis, leads to histone hyperacetylation (Galdieri and Vancura, 2012; McDonnell et al., 2016).

Recent studies showed that fatty acid metabolism regulates histone acetylation by modulating histone deacetylation. For example, several long-chain free fatty acids including myristic, oleic and linoleic acids have been shown to bind to SIRT6 and induce up to a 35-fold increase of its activity toward H3K9ac (Feldman et al., 2013). Although oleic acid has no effect on the purified SIRT1 activity, intracellular elevated oleic acid stimulates the deacetylase activity of SIRT1 by inducing its Ser434 phosphorylation via the cAMP/PKA signaling pathway (Lim et al., 2013). Some short-chain fatty acids, i.e., butyric acid, valproic acid function as HDACs inhibitors to increase histone acetylation. The product of fatty acid oxidation, β-hydroxybutyrate (βOHB) is an endogenous and specific inhibitor of class I HDACs (Shimazu et al., 2013), linking the activity of histone deacetylases to ketone body metabolism. During fasting, the levels of β-hydroxybutyrate can rise up to 1 mM as the byproduct of fatty acid oxidation in the liver, which inhibits the activity of class I HDACs to increase global H3K9 and H3K14 acetylation (Shimazu et al., 2013). Moreover, β-hydroxybutyrate specifically induces H3K9 acetylation at the promoters of oxidative stress resistance genes, *Foxo3a* and *Mt2* to activate their transcription and protect cells against oxidative stress (Shimazu et al., 2013). In contrast to the situation that high calorie diets tend to inhibit the deacetylase activity of sirtuins via NAD^+^, calorie restriction conditions could inhibit the deacetylase activity of class I HDACs by β-hydroxybutyrate.

Histone methylation is regulated by lipid metabolism (Ye et al., 2017). Cells lacking phosphatidylethanolamine (PE) methyltransferases interrupt SAM homeostasis, leading to hypermethylation of histone modifications: H3K4me3, H3K36me3, and H3K79me3 and misregulation of gene expression (Ye et al., 2017).

### Direct Modulation of Epigenetic Modifications by Metabolic Enzymes

Recent studies showed that metabolic enzymes also directly regulate chromatin modifications independent of their produced metabolites, which is one of non-metabolic functions of metabolic enzymes (Yu and Li, 2017). The typical example is pyruvate kinase PKM2, which is specifically upregulated in tumors. Upon epidermal growth factor (EGF) receptor activation, PKM2 functions as a protein kinase to phosphorylate histone H3 on threonine 11 (H3T11) (Yang et al., 2012). PKM2-catalyzed H3T11 phosphorylation displaces histone deacetylase 3 (HDAC3) from the promoters of *CCDN1* and *MYC*, leading to increased H3K9 acetylation, induced transcription of *CCDN1* and *MYC*, and brain tumorigenesis (Yang et al., 2012). We have previously shown that the yeast homolog of PKM2, pyruvate kinase 1 (Pyk1) phosphorylates H3T11 and its activity is activated by glucose-derived serine, which in turn represses the transcription of *PYK1* (Li et al., 2015; Yu and Li, 2017). This feedback regulation the expression of pyruvate kinase could confer cells resistance to oxidative stress (Yu et al., 2017). PKM2 and hexokinase 1 (HK1) have also been reported to phosphorylate histones H1 and H2A (Adams et al., 1991; Ignacak and Stachurska, 2003). However, little is known about the phosphorylation sites and the biological functions of H1 and H2A phosphorylation remain elusive.

Some metabolic enzymes can phosphorylate non-histone proteins. An unbiased quantitative phosphoproteomic approach revealed that PKM2 can phosphorylate a total of 974 proteins, including mammalian target of rapamycin (mTOR) inhibitor AKT1 substrate 1 (AKT1S1) (He et al., 2016). PKM2 phosphorylates AKT1S1 at serine 202 and 203 to enhance its binding to 14-3-3, leading to mTOR activation, autophagy inhibition and accelerated cancer cell growth (He et al., 2016). Moreover, PKM2 activates the transcription factor, STAT3 by phosphorylating its Tyr305, which promotes the transcription of genes required for tumor cell proliferation (Gao et al., 2012; Yu and Li, 2017). Phosphoglycerate kinase 1 (PGK1) and pyruvate dehydrogenase kinase 1 (PDHK1) also function as protein kinases (Yu and Li, 2017). Mitochondrial PGK1 phosphorylates PDHK1 at T338, which activates PDHK1 to phosphorylate and inhibit PDC, resulting in reduced oxidation of pyruvate to CO_2_ and reactive oxygen species (ROS) production in the mitochondria and increased lactate production (Li et al., 2016). Moreover, PGK1-mediated PDHK1 phosphorylation at T338 promotes cancer cell proliferation and tumorigenesis and PDHK1 phosphorylation level correlates with glioblastoma prognosis (Li et al., 2016).

Certain glycolytic enzymes regulate histone gene expression or histone cleavage. Nuclear glyceraldehyde-3-phosphate dehydrogenase (GAPDH) and lactate dehydrogenase (LDH) promote histone *H2B* transcription by forming the OCA-S coactivator complex with octamer binding protein (Oct-1) (Zheng et al., 2003). Glutamate dehydrogenase 1 (Gdh1), an important enzyme in glutamine synthesis, has been reported to function as a histone H3-specific protease, which regulates histone modifications by clipping the N-terminal residues of H3 (Mandal et al., 2013).

### Modulation of Epigenetic Modifications by Oxygen

The demethylation reaction is a redox reaction and it has been well established that histone demethylation is regulated by oxygen availability. Hypoxia has been reported to increase H3K4me3 in mammalian cells by inhibiting the activity of the oxygen-dependent H3K4 demethylase JARID1A (Zhou et al., 2010). Generally, low oxygen inhibits histone demethylation through the following mechanisms: lack of oxygen as a substrate for demethylases; increased protein levels of oxygen sensor hypoxia-inducible factor (HIF) and associated histone modifying enzymes (Melvin and Rocha, 2012); selective synthesis of L2-HG, the enantiomer of 2-HG, which is also an competitive inhibitor of α-KG-dependent demethylases (Intlekofer et al., 2015).

## Regulation of the Specificity of Epigenetic Modifications by Metabolism

To regulate the activity of epigenetic modifiers, the concentration of metabolites as substrates should be well above the enzymatic Km, which refers to the substrate concentration that produces half-maximal velocity and is used to measure the binding affinity of enzymes to their substrates. In fact, for most chromatin modifiers with the exception of protein kinases, their measured Km is within the range of physiological concentrations of metabolites. Therefore, their enzymatic activity could be regulated by the availability of substrates and cofactors derived from the metabolic pathways. However, accumulating evidence showed that perturbations in metabolites availability only influence certain types of chromatin modifications. For example, increasing the cellular SAM levels by methionine and folate amendment specifically increased H3K4me2/me3 but not H3K79me3 (Sadhu et al., 2013). How is the specificity of epigenetic modifications determined by metabolism? Why are only a few specific chromatin regions sensitive to metabolism alterations despite the genome-wide presence of similar chromatin markers? In principle, there are two potential determinants: differential enzymatic characteristics and local production of metabolites.

### Differential Enzymatic Characteristics and the Specificity of Epigenetic Modifications

Different chromatin modifying enzymes have different kinetic parameters, such as Km. This different Km implied that a change in substrate concentrations would differentially influence the enzymatic activity and there is a hierarchy of sensitivity to nutritional limitations. The Km of H3K4 methyltransferase MLL1 for SAM is 10.4 μM whereas that of H3K27 methyltransferase EZH2 is 1.64 μM, which makes MLL1-catalyzed H3K4me3 more sensitive to changes in the intracellular SAM levels than EZH2-catalyzed H3K27me3 (Mentch et al., 2015). Indeed, methionine restriction results in dramatic reduced H3K4me3 as a consequence of decreased SAM (Mentch et al., 2015; Dai et al., 2018). The specific response of H3K4 methylation to SAM level changes has also been observed in yeast cells (Sadhu et al., 2013). H3K79 methyltransferase Dot1 has a lower Km than that of the H3K4 methyltransferase Set1, which makes H3K79 methylation resistant to changes in one-carbon metabolism. However, increasing the Km of Dot1 for SAM by mutating its G401 makes H3K79 dimethylation significantly affected by folate deficiency (Sadhu et al., 2013). A similar situation could also occur for histone acetylation specificity. In human cells, the Km of Gcn5 and P/CAF are 0.62 μM and 0.64 μM, respectively, while the Km of p300 for acetyl-CoA is 6.7 μM (Fan et al., 2015). Given the intracellular acetyl-CoA is 2–20 μM, the difference in their Km values may lead to differential response of these three enzymes and hence substrate acetylation to acetyl-CoA availability.

### Local Production of Metabolites and the Specificity of Epigenetic Modifications

Instead of causing global chromatin changes, most nutritional alterations affect chromatin modifications on specific locus, which cannot be explained by the differential kinetic properties of modifying enzymes. For example, elevated acetyl-CoA levels have been shown to increase histone acetylation only at a subset of genes, i.e., growth-promoting genes (Cai et al., 2011). In addition, the difference in kinetic properties of modifying enzymes does not work very well for metabolites that are not stable and cannot easily cross cellular membranes, i.e., acetyl-CoA. It is likely that these metabolites are compartmentalized and locally produced. The “local production and local consumption” model could better explain the gene context-dependent specificity by metabolites. In this model, some metabolites-generating enzymes may form a complex with epigenetic modifiers and/or transcription factors, which can be recruited to specific locus by chromatin-associated factors, thereby determining the locus-specific modifications. PDC has been reported to form a novel complex with PKM2, p300, and aryl hydrocarbon receptor (AhR), which is recruited to AhR target gene, *cytochrome P4501A1* (*CYP1A1*) in a AhR-dependent manner (Matsuda et al., 2016). PDC acts together with PKM2 to provide localized acetyl-CoA for p300 to acetylate H3K9 at *CYP1A1* enhancer and activate its transcription (Matsuda et al., 2016). In addition to acetyl-CoA, the SAM synthesis enzyme, methionine adenosyltransferase II (MATII) interacts with the H3K9 methyltransferase SETDB1 within a protein complex to regulate H3K9 methylation at *COX2* and repress its transcription (Katoh et al., 2011; Kera et al., 2013). We have previously found that SAM synthetases (Sam1 and Sam2), serine metabolic enzymes (Ser33 and Shm2) and pyruvate kinase Pyk1 form a novel complex called SESAME, which interacts with Set1 methyltransferase complex to promote Set1-catalyzed H3K4 methylation at pyruvate kinase 1 (*PYK1*) (Li et al., 2015; Yu et al., 2017). There is also a report about regulation of H3K36 methylation by locally produced fumarate upon DNA damage. The fumarate-producing enzyme, fumarase is recruited to DNA double-strand break (DSB), where it interacts with histone variant H2A.Z after exposure to ionizing radiation (Jiang et al., 2015). The locally produced fumarate then inhibits the activity of demethylase KDM2B, resulting in enhanced H3K36 dimethylation at DSB sites (Jiang et al., 2015).

NAD^+^ is also produced in the same way to determine the specificity. The NAD^+^-producing enzymes GAPDH and LDH have been reported to translocate into the nucleus and interact with transcription factors and chromatin modifiers (Zheng et al., 2003; Berger et al., 2005; Castonguay et al., 2014). Nuclear GAPDH and LDH form an OCA-S coactivator complex, which interacts with the octamer binding protein (Oct-1) and is recruited by Oct-1 to *H2B* promoter, where locally produced NAD^+^/NADH regulates *H2B* transcription (Zheng et al., 2003). Nuclear LDH also interacts with SIRT1 and supplies NAD^+^ to SIRT1 to enhance its activity to deacetylate histones in human hepatocytes, which might help cells resist oxidative stress (Castonguay et al., 2014). The yeast GAPDH interacts with Sir2 in the nucleus, providing local NAD^+^ to activate Sir2 and promote gene silencing (Ringel et al., 2013). Altogether, these examples suggest that local generated metabolite pools have biological significance by dictating specific epigenetic modifications.

### Signaling Pathways That Regulate Metabolism and Epigenetic Modifications

The epigenetic landscape plays a crucial role in cellular adaptation to changes in nutrient availability and utilization. But the remaining question is how nutrient changes are transduced to alterations in epigenetic modifications? In addition to intermediary metabolites, such as acetyl-CoA and SAM, which function as an indicator to reflect the cells’ potential to generate energy (Fan et al., 2015), there are several other nutrient and bioenergetic sensors including phosphoinositide 3-kinase (PI3K)/Akt, AMP-activated protein kinase (AMPK), mammalian target of rapamycin (mTOR). These molecules sense nutritional changes and transduce these changes to chromatin modifications.

### PI3K/AKT Pathway

**FIGURE 5:**
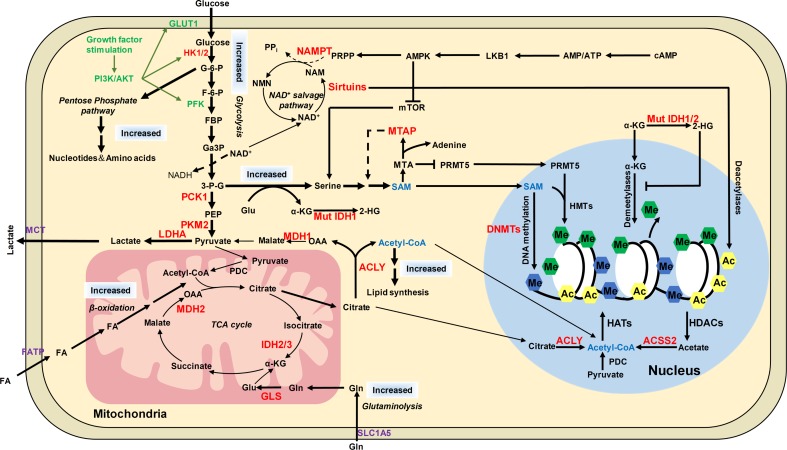
The distinct metabolic pathways in cancer cells and their connection to epigenetic modifications. Cancer cells have increased glucose and glutamine uptake, leading to accelerated glycolysis and biomass accumulation. PI3K/AKT is activated by growth factors to regulate glycolysis and acetyl-CoA generation. AKT induces the expression of GLUT1 to increase glucose uptake and phosphorylates HK1 and PFK to enhance aerobic glycolysis. AKT phosphorylates ACLY to increase acetyl-CoA production. Increased lactate dehydrogenase (LDH) activity and decreased pyruvate dehydrogenase (PDC) activity result in increased lactate export, attenuated TCA cycle and diversion of glycolysis to pentose phosphate pathway. ACLY, ACSS2 and PDC contribute to nuclear acetyl-CoA production and subsequent histone acetylation. Glycolysis is increased in cancer cells with NAD^+^ converted to NADH, leading to reduced NAD^+^/NADH ratio and downregulated activity of sirtuins, which results in histone hyperacetylation and dysregulation of gene expression. Serine and one-carbon metabolism is also accelerated in cancer cells to produce SAM, which is controlled by LKB-AMPK-mTOR pathway. α-KG is primarily produced by transamination of glutamate derived from glutamine in cancer cells. Mutations of IDH1/2 lead to accumulation of 2-HG, which increases histone methylation by inhibiting α-KG dependent enzymes. The potential targets for anti-cancer therapy were labeled in red color. ACSS2, acetyl-CoA synthetase short chain family member 2; AMPK, AMP-activated protein kinase; F-6-P, fructose-6-phosphate; FBP, fructose-1,6-biphosphate; GA3P, glyceraldehyde-3-phosphate; G-6-P, glucose-6-phosphate; Gln, glutamine; Glu, glutamate; HK1/2, hexokinase 1/2; LDHA, lactate dehydrogenase; MCT1, monocarboxylate transporter 1; MCT4, monocarboxylate transporter 4; MDH1, malate dehydrogenase 1; Mut IDH1/2, mutated isocitrate dehydrogenase 1 and 2; MTA, 5′-methylthioadenosine; MTAP, MTA phosphorylase; NAM, nicotinamide; NAMPT, nicotinamide phosphoribosyltransferase; NMNATs, nicotinamide mononucleotide adenylyltransferases; PCK1, phosphoenolpyruvate carboxykinase 1; PDC, pyruvate dehydrogenase complex; PEP, phosphoenolpyruvate; PFK, phosphofructokinase; PI3K, phosphoinositide 3-kinase; PKM2, pyruvate kinase M2; PRMT5, arginine methyltransferase 5; PRPP, phosphoribosyl pyrophosphate; α-KG, α-ketoglutarate; 2-HG, 2-hydroxyglutarate; 3-P-G, 3-phosphoglycerate; ACLY, ATP-citrate lyase.

Phosphoinositide 3-kinase (PI3K)/Akt pathway is a critical signaling cascade in response to growth factor stimuli and reflects in acetyl-CoA and histone acetylation changes (Lee et al., 2014). The PI3K/Akt pathway is frequently activated in cancer cells, i.e., prostate cancer and the activity of Akt correlates with histone acetylation levels (Lee et al., 2014). The PI3K/Akt cascade is initiated with PI3K activation by binding of extracellular growth factors to cell membrane receptor tyrosine kinases (RTKs). Activated PI3K phosphorylates the membrane phosphatidylinositol lipids, which then recruit and activate the downstream effector kinase Akt. Akt stimulates acetyl-CoA production and histone acetylation by the following mechanisms (**Figure 5**; Kim, 2018): (1) Akt upregulates the expression of glucose transporter GLUT1 to promote glucose uptake; (2) Akt accelerates aerobic glycolysis by phosphorylating and activating glycolytic enzymes hexokinase (HK1) and phosphofructokinase 1/2 (PFK1/2); (3) Akt phosphorylates and activates ACLY to increase the production of acetyl-CoA. Cancer cells activate Akt to maintain a high level of nuclear acetyl-CoA, preventing histone hypoacetylation from fluctuations in nutrient availability.

### AMPK Pathway

AMP-activated protein kinase (AMPK) is an AMP-sensitive protein kinase that functions as an energy sensor to regulate mitochondrial biogenesis in cells (Canto et al., 2009). AMPK signaling is activated by decreased ATP synthesis and elevated cellular AMP/ATP ratio when cells are grown under glucose restriction conditions. The activated AMPK then increase NAD^+^/NADH ratio, which subsequently activates the deacetylation activity of sirtuins (Canto et al., 2009). Moreover, AMPK directly phosphorylates SIRT1 at T344, which activates SIRT1 by dissociating SIRT1 from its endogenous inhibitor DBC1 (Lau et al., 2014). In turn, SIRT1 deacetylates and inactivates the p53 tumor suppressor. Inhibition of AMPK by Compound C leads to increased p53 acetylation (Lau et al., 2014). In addition, AMPK may control the homeostasis of acetyl-CoA and hence histone acetylation. AMPK and its homolog Snf1 in yeast have been shown to phosphorylate and inhibit the activity of acetyl-CoA carboxylase (ACC), leading to increased acetyl-CoA and consequently hyperacetylation of histones (Zhang et al., 2013).

### mTOR Pathway

The mammalian target of rapamycin complex (mTORC) participates in signal transduction pathways that transduce growth factor signals and nutrients signals to transcription and translational control, thus determining cell growth and proliferation status (Faivre et al., 2006). mTOR is a serine/threonine kinase that interacts with different proteins to form two distinct complexes: mTOR complex 1 (mTORC1) and mTOR complex 2 (mTORC2), which share catalytic subunits mTOR, MLST8, and DEPTOR. The mTORC1 is sensitive to rapamycin but the mTORC2 signaling is insensitive to nutrients (Vadla and Haldar, 2018). mTORC1 is constitutively activated in cancer cells, which regulates protein synthesis and stability to provide survival and proliferation advantages over normal cells (Guertin and Sabatini, 2007). The mTORC1 activity is controlled by liver kinase B1 (LKB1)/AMPK signaling cascade (**Figure 2**; Kottakis et al., 2016). LKB1, which is a kinase that activates AMPK, is a tumor suppressor inactivated in a range of sporadic cancers, including pancreatic adenocarcinoma (Su et al., 1999). Inactivation of LKB1 led to activation of mTORC1 pathway, resulting in upregulated serine biosynthesis, elevated SAM generation and increased expression of DNA methyltransferases (DNMTs), which in turn, enhanced global DNA methylation and transcriptional silencing of retrotransposons (Kottakis et al., 2016; **Figure 2**).

A recent report showed that mTORC2 but not mTORC1 signaling pathway regulates histone acetylation H3K56ac (Vadla and Haldar, 2018). mTORC2 promotes H3K56ac at the promoters of glycolytic genes by interfering with the recruitment of SIRT6 and is required for transcription of these genes, which mediates metabolic reprogramming in glioma (Vadla and Haldar, 2018).

## Epigenetic Regulation of Cell Metabolism

### Epigenetic Regulation of Metabolic Gene Expression

It is noteworthy that there is a bidirectional relationship between epigenetic modifications and metabolism. On one hand, cell metabolites and metabolic enzymes modulate epigenetic modifications; on the other hand, epigenetic changes at metabolic genes regulate the transcription of genes involved in metabolism, which eventually affects cell metabolism (Yu and Li, 2017). The expression of type II hexokinase (HKII), a key enzyme in glycolysis, is up-regulated in liver cancer by DNA hypomethylation, which could accelerate glycolytic flux in cancer cells (Goel et al., 2003; Johnson et al., 2015). The methyltransferase G9a activates the transcription of genes involved in serine-glycine biosynthetic pathway by catalyzing H3K9 monomethylation (Ding et al., 2013). G9a inactivation reduces intracellular serine and its downstream metabolites, causing cancer cell death (Ding et al., 2013; **Figure 2**). Acetyl-CoA treatment upregulates the expression of metabolic gene *SHM2* by increasing histone acetylation (Cai et al., 2011). Histone demethylase LSD1 represses the expression of two gluconeogenic genes, fructose-1,6-bisphosphatase (FBP1), and glucose 6-phosphatase (G6Pase) by demethylating H3K4me2 at their promoters (Pan et al., 2013). Histone acetyltransferase NuA4 has been reported to contribute to phospholipid homeostasis by regulating the expression of inositol-3-phosphate synthase gene, *INO1* (Dacquay et al., 2017). It remains unclear whether NuA4 regulates *INO1* transcription via histone acetylation.

The bidirectional regulation between metabolism and epigenetic modifications could lead to a feedback control of cell metabolism: intracellular metabolism perturbations change epigenetic modifications at metabolic genes, which influences the transcription of these genes and metabolic pathways. The feedback regulation of metabolism could enable cells to respond to changes in microenvironment in a prompt and accurate way. Glucose metabolism has been shown to stimulate pyruvate kinase 1 (Pyk1)-catalyzed H3T11 phosphorylation, which represses the transcription of *PYK1*, which leads to reduced glycolytic flux and increased resistance to oxidative stress (Li et al., 2015).

### Modifications of Metabolic Enzymes

Another way to regulate cell metabolism is modifying metabolic enzymes, which may affect their activity, stability and/or subcellular localization.

Acetylation is an important way to control the activity of many metabolic enzymes. For example, a number of mitochondrial proteins have been reported to be inactivated by acetylation to suppress mitochondrial functions (Hirschey et al., 2010). These proteins include enzymes involved in TCA cycle (IDH2 and SDH), complex I in the electron transport chain and superoxide dismutase (MnSOD). Moreover, acetylation of PDC and enzymes in fatty acid oxidation reduces the entry of acetyl-CoA into the TCA cycle (Kinnaird et al., 2016). As acetylation is a reversible process, deacetylation is also an important way to regulate protein activity. These mitochondrial enzymes are deacetylated by SIRT3 to relieve mitochondrial suppression (Kinnaird et al., 2016). Acetylation is required to enhance the activity of the glycolytic enzyme phosphoglycerate mutase-1 (PGAM1) and SIRT1-mediated deacetylation reduces its catalytic activity (Hallows et al., 2012). Under glucose restriction conditions, SIRT1 levels are dramatically increased, which deacetylates and inactivates PGAM1. The mitochondrial acetyl-CoA synthetase ACSS2 is deacetylated and activated by SIRT1 under low-nutrient conditions, providing a compensatory way to produce acetyl-CoA (Hallows et al., 2006).

Acetylation also controls the stability of some metabolic enzymes. ACLY is deacetylated by SIRT2 to become unstable (Lin et al., 2013). High glucose has been shown to induce ACLY acetylation at lysine 540, 546, and 554 by p300/CBP-associated factor (PCAF) acetyltransferase to prevent ACLY from ubiquitylation and degradation, which in turn promotes *de novo* lipid synthesis and tumor growth (Lin et al., 2013). Most importantly, the acetylation levels of ACLY are elevated in lung cancers, implying that ACLY acetylation could be a potential biomarker for lung cancer diagnosis (Lin et al., 2013).

Acetylation promotes the translocation of several glycolytic enzymes to the nucleus where they function as transcriptional regulators. For example, PKM2 is acetylated by p300, which promotes its translocation into the nucleus and contributes to tumor cell proliferation and tumorigenesis (Lv et al., 2013). The translocation of GAPDH to the nucleus also requires PCAF-mediated acetylation at its three lysine residues (lysine 117, 227, 251; Ventura et al., 2010).

Metabolic enzymes also undergo other modifications, i.e., phosphorylation, acylation, etc. Upon DNA damage, nuclear ACLY is phosphorylated, which enhances its ability to synthesize the nuclear acetyl-CoA pool and increases histone acetylation required for efficient double-strand break repair by homologous recombination (Sivanand et al., 2017). Huang et al. found that p300 functions as a lysine 2-hydroxyisobutyryl transferase to regulate glycolysis in response to nutritional cues (Huang et al., 2018). p300 catalyzes lysine 2-hydroxyisobutyrylation on glycolytic enzymes, i.e., ENO1 to enhance their catalytic activity and knockout of p300 leads to impaired glycolysis and reduced growth of cancer cells when cultured in glucose-depleted medium (Huang et al., 2018).

### Modifications as Storage for Metabolites

In addition to modulate the activity of metabolic enzymes and the expression of metabolic genes, epigenetic modifications also serve as the storage for metabolites. The typical example is recycling the acetyl group from acetylated proteins in the form of acetate by class I and II HDACs. ACSS2 then synthesizes acetyl-CoA from acetate. Based on the potential acetylation sites, yeast histones can store up to 65-fold more acetyl groups than acetyl-CoA and mammalian proteins can store ∼100-fold more acetyl moieties than free acetyl-CoA (Fan et al., 2015). This implied that under carbon limited conditions, cells can potentially replenish acetyl-CoA pools by recycling the acetyl group from acetylated proteins. This recycled acetyl group from acetylated proteins may not be sufficient to support the normal metabolic activity under nutrient rich conditions but may be required for immediate survival when cells consume acetyl-CoA at a lower rate under metabolic stress (Fan et al., 2015). Histone acetylation and deacetylation reactions may also regulate other metabolic processes, i.e., intracellular pH balance. McBrian et al. (2013) reported that under low pH, global histone deacetylation is increased with a large amount of acetate generated. The acetate anions produced by HDACs are then co-exported out of cells along with protons, thereby preventing further decrease of intracellular pH (McBrian et al., 2013). This suggests a role for histone acetylation and deacetylation in regulating intracellular pH. Sirtuins remove protein acetylation by transferring the acetyl group to NAD^+^, yielding O-acetyl-ADP-ribose, which is required for Sir3 to bind to telomeres, enhancing transcription silencing at telomere proximity regions (Ehrentraut et al., 2010).

Histone demethylation results in hydroxylation of the enzymatic substrate to generate formaldehyde, which is an endogenous protein and DNA cross-linking agent. Formaldehyde can be detoxified by converting to formate, which then functions as one-carbon unit to fuel nucleotide biosynthesis (Burgos-Barragan et al., 2017). This endogenously produced formaldehyde may provide one-carbon unit to support the growth of cells that cannot use serine, which is the predominant source of one-carbon unit under most circumstances (Burgos-Barragan et al., 2017). All α-KG-dependent dioxygenases use oxygen and α-KG as substrates and release succinate and CO_2_ as byproducts. α-KG and succinate are intermediary metabolites of the TCA cycle. Whether succinate can be reused in TCA cycle needs to be explored.

## Conclusion

Cancer cells have distinct metabolic pathways and epigenetic landscapes with their normal counterparts, which contribute to tumorigenesis (**Figure 5**). The metabolic and epigenetic landscape rewiring not only facilitates cancer cells adapt to nutritional changes in microenvironment but also can become potential targets for anti-cancer therapy. Most metabolic enzymes that regulate epigenetic modifications are upregulated in cancers and some have become the target for cancer therapy (Yu and Li, 2017). The first-in-class inhibitor of mutant IDH2 was approved by FDA for treatment of acute myeloid leukemia (AML) in 2017 (Szczuka et al., 2017). Many other compounds are under development or investigation. For example, ACLY, ACSS2, and PDC have been considered as potential molecular targets for anti-cancer therapy due to their functions in nuclear acetyl-CoA production and histone acetylation. Several drugs targeting these three proteins are under preclinical or clinical investigation including ACLY inhibitors (SB-204990 and BMS-303141 in preclinical stage, ETC-1002 in phase II clinical trial and hydroxycitrate in phase IV clinical trial), ACSS2 inhibitor (*N*-(2,3-di-2-thienyl-6-quinoxalinyl)-*N′*-(2-methoxyethyl)urea in preclinical stage) and PDC activator dichloroacetate (Kinnaird et al., 2016). The key enzyme in serine synthesis and SAM metabolism, PHGDH is a promising candidate and two PHGDH inhibitors, NCT-502 and NCT-503, have been shown to reduce tumor cell growth (Pacold et al., 2016; Rohde et al., 2018). Glycolytic enzymes have also become targets for cancer therapy and the corresponding compounds include “hexokinase 2 (HK2) (2-deoxyglucose, 3-bromopyruvic acid, trastuzumab, and lonidamine), PKM2 (substituted N,N*′*-diarylsulfonamides, substituted thienol[3,2]pyrrole[3,2d]pyridazinone scaffold, TEPP-46, DASA-58, cisplatin, and docetaxel), and lactate dehydrogenase (LDHA) (gossypol, FX11, galloflavin, FK866, oxamate, and paclitaxel) (Yu and Li, 2017)”. NAMPT has become an attractive target for cancer treatment due to its role in controlling sirtuins’ activity in tumors, and its inhibitor, APO866 has been used in Phase II clinical trials (Khan et al., 2007). Other metabolism inhibitors, such as glutaminase inhibitor CB-839 and 3-Bromopyruvate are currently in clinical trials (Szczuka et al., 2017). These agents inhibit tumor growth may not only by affecting metabolism but also changing epigenetic modifications. Elucidation the connection between metabolism and epigenome will not only shed lights on understanding cancer pathogenesis but also help develop anti-cancer therapy that of more efficiency and high specificity. As described previously, the MTAP-deficient cancer cells are more sensitive to the PRMT5 inhibitor (Kryukov et al., 2016). It is conceivable that the combined use of drugs targeting metabolic pathways and epigenetic machinery may exhibit synergistic effect on anti-cancer therapy.

Although tremendous progress has been made in understanding the connection between cancer metabolism and epigenetics, there are several open and outstanding questions need to be addressed. Firstly, the list of metabolic enzymes present in the nucleus is continually expanding. Understanding their roles in the nucleus is critical to elucidate the connection between metabolism and epigenetic regulation. However, since many enzymes lack a canonical nuclear localization sequence (NLS), it remains unclear how they enter into the nucleus. Moreover, many nuclear metabolic enzymes function within a complex via interaction with other proteins. Thus, characterizing their interaction partners in the nucleus will help us better understand their non-metabolic functions. Secondly, numerous transcriptomic studies showed that the effect of metabolites on gene transcription was specific rather than global. For example, Cai et al. (2011) showed that a rise of global acetyl-CoA only increases histone acetylation at growth-promoting genes. This specific effect was also observed in modifications of non-histone proteins. Modulating the availability of acetyl-CoA only affects the acetylation of a subset of cellular proteins. Although we described two possible mechanisms to explain how metabolism determines the specificity of epigenetic modifications, it is difficult to examine these two mechanisms given the fact that it is impossible to measure the accurate concentrations of individual metabolites, especially their local concentrations with our current technology. Lastly, as depletion of critical metabolites or disturbing cellular metabolism may affect cell viability and proliferation, the experimental challenge to study cellular metabolism is to uncouple their direct effects on chromatin from their secondary effects on viability and proliferation.

## Author Contributions

XY and SL conceptualized the study and wrote, reviewed, and edited the manuscript. XY, RM, YW, YZ, and SL wrote the draft.

## Conflict of Interest Statement

The authors declare that the research was conducted in the absence of any commercial or financial relationships that could be construed as a potential conflict of interest.
